# Human Behavior Analysis Using Intelligent Big Data Analytics

**DOI:** 10.3389/fpsyg.2021.686610

**Published:** 2021-07-06

**Authors:** Muhammad Usman Tariq, Muhammad Babar, Marc Poulin, Akmal Saeed Khattak, Mohammad Dahman Alshehri, Sarah Kaleem

**Affiliations:** ^1^Abu Dhabi School of Management, Abu Dhabi, United Arab Emirates; ^2^Department of Computer Science, Allama Iqbal Open University, Islamabad, Pakistan; ^3^Department of Computer Science, Quaid-i-Azam University, Islamabad, Pakistan; ^4^College of Computers and Information Technology, Taif University, Taif, Saudi Arabia; ^5^Department of Computing and Technology, Iqra University, Karachi, Pakistan

**Keywords:** human behavior, big data, artificial intelligence, Apache Spark, analytics

## Abstract

Intelligent big data analysis is an evolving pattern in the age of big data science and artificial intelligence (AI). Analysis of organized data has been very successful, but analyzing human behavior using social media data becomes challenging. The social media data comprises a vast and unstructured format of data sources that can include likes, comments, tweets, shares, and views. Data analytics of social media data became a challenging task for companies, such as Dailymotion, that have billions of daily users and vast numbers of comments, likes, and views. Social media data is created in a significant amount and at a tremendous pace. There is a very high volume to store, sort, process, and carefully study the data for making possible decisions. This article proposes an architecture using a big data analytics mechanism to efficiently and logically process the huge social media datasets. The proposed architecture is composed of three layers. The main objective of the project is to demonstrate Apache Spark parallel processing and distributed framework technologies with other storage and processing mechanisms. The social media data generated from Dailymotion is used in this article to demonstrate the benefits of this architecture. The project utilized the application programming interface (API) of Dailymotion, allowing it to incorporate functions suitable to fetch and view information. The API key is generated to fetch information of public channel data in the form of text files. Hive storage machinist is utilized with Apache Spark for efficient data processing. The effectiveness of the proposed architecture is also highlighted.

## Introduction

Intelligent big data analysis is an evolving pattern in the age of data science, big data, and artificial intelligence (AI). Data has been the backbone of any enterprise and will do so moving forward. Storing, extracting, and utilizing data has been key to any operations of a company (Little and Rubin, 2019). When there were no interconnected systems, data would stay and be consumed in one place. With the onset of Internet technology, the ability and requirement to share and transform data have been exploited (Maceli, 2020). With the spread of social media, the nature of data has changed. Social media can consist of billions of users who continuously provide their digital traces with incredible velocity (Kumar et al., 2018). As the data comes from many sources and in an unstructured format, it is not easy to handle in traditional relational databases. The need for handling unstructured data gives birth to another type of data called big data, which is unstructured, semi-structured, and unpredictable (Iqbal et al., 2020). This data is created real-time, and the amount of data is increasing daily. The data generated from these social media sites can take the form of text, images, videos, and documents. Only structured data can be processed and stored using an RDBMS. Big data is used to process data with a huge volume that is not possible to process using old database techniques and traditional relational databases, within an acceptable processing time.

Big data is characterized by a large volume of data with a large variety and higher velocity (Wang et al., 2020). Data generated moves through cables, either TV or internet, and data on local TV cables broadcast with large volume, variety, and velocity. The amount of data generated every day in the world is increasing exponentially. The rate of data growth is surprising, and this data comes at a speed, with variety (not necessarily structured), and contains a wealth of information that can be key for gaining an edge in competing businesses. The ability to analyze this massive amount of data brings a new era of innovation, productivity growth, and consumer surplus. “Big data is the term for a collection of data sets so large and complex that it becomes difficult to process it using traditional database management tools or data processing applications” (Cui et al., 2020). The challenges include capturing, curating, storing, searching, sharing, transferring, analyzing, and visualizing this data. This section discusses the related literature.

Big data is described with 5V’s instead of 3V (volume, velocity, and variety) and included veracity and value (Grover et al., 2020). The widely known big data examples are social networking sites, such as Facebook, YouTube, Dailymotion, Google, and Twitter (Drosos et al., 2015). These sites receive a tremendous amount of data regularly with different variety, velocity, and veracity. The data include value as well. As the number of users increases, the amount of data also increases day by day. Users and data both keep growing on these sites, and this amount of data is a big challenge for owners and companies. This data contains all useful information that needs to be processed in a concise period. To generate more revenue and increase sales, the companies need the processed and analyzed data. The analysis of this data is not possible through relational or traditional database systems within a given time frame as the resources of this traditional system are not sufficient to accomplish processing and storing this huge amount of data; hence, Hadoop comes into the existence for fulfilling this need. In recent years, a large amount of unstructured data is generated from social media sites, such as Facebook, Twitter, Google, and some Dailymotion forums in the form of images, text, videos, and documents, to access and analyze this type of data, this work is best for practicing in the entire field (Xia et al., 2018). Twitter and Facebook are some of the most famous social media platforms, and the companies find that it is very crucial for obtaining customer feedback and maintaining goodwill.

Dailymotion is one of the best video-sharing social media websites. It is a viral platform that publishes community feedback through its videos and comments, likes, dislikes, published videos, and subscriber information for a particular channel (Stieglitz et al., 2018). The analysis of this type of data is important for acquiring knowledge about users, categories, and interests of users. Most of the production companies have their channels to share daily their movie trailers for getting user feedback before releasing them to the general public. Furthermore, individual users upload their videos to get more subscribers and views. These data points are critical for owners to analyze data to understand the views and feelings of customers about their video and service. Dailymotion has billions of users, who watch hours of videos on their site and generate a massive amount of views (Carlinet et al., 2012). It is estimated that more than a hundred hours of videos are watched per minute, and this amount is increasing day by day. To analyze such a huge amount of data, relational databases are not applicable. Users can use this data to understand how much their marketing program is effective. They can check their view counts and subscribers based on the date range that will show them the peak and downtime of views in a particular time. This will also help to check social trends and behavior of people over time (Lee and Kotler, 2011). For example, users can check how many views their videos have received and how much people have liked their video or product. They can also analyze likes and dislikes from the diverse nature of people around the world.

In this research, we utilized Apache Spark to process datasets of social media. Apache Spark is a parallel and distributed platform that overcomes the challenges faced by the traditional processing mechanisms. The main objective of the project is to demonstrate the use of Apache Spark parallel and distributed framework technologies with other storage and processing mechanisms. The social media data generated from Dailymotion is taken under consideration in this article.

## Literature Review

A framework is proposed for computing fast and reliable data analysis and mining feedbacks (Rodrigues and Chiplunkar, 2018). They give the real-time Twitter data input in the framework for getting the results of the analysis to generate fast feedback through sentiment analysis. As per Rodrigues and Chiplunkar (2018), the accuracy of data analysis results is essential, and the Hadoop framework provides more than 84% of results when data is produced from social media. Twitter data is one of the largest social media networks where data is increasing daily (Rodrigues et al., 2017). The researcher used data analysis using the “InfoSphere Big Insights” tool, which is very suitable for enterprise companies to use the power of Hadoop in real-time data analysis. The data analytics in Blomberg’s work are beneficial for companies to collect customer feedback and details of current trends (Blomberg, 2012). Many big companies, such as Airlines and some other related companies, use these analytics to reach their customers based on their feedback. For crime investigation, cyber-crime people search individuals who have committed the crime.

An architecture is proposed for the sentiment analysis of Twitter by using Hadoop components simply called the ecosystem of Hadoop (Mahalakshmi and Suseela, 2015). It provides the mechanism of Tweets analysis on clusters of Hadoop. It also provided a complete pictorial form of data from various users and their tweets. Recently, newspapers are not read as often and people use television and the internet for most sources of information. Furthermore, many tasks are now done online, such as trading stocks. Buying and selling of shares can be done through the internet from a single laptop or even through mobile (Khan and Khan, 2018). Customers watch every second trend of the stock exchange through their mobile. In this way, they are aware of market fluctuations. To predict the market, Hadoop is used for the analysis of real-time data. The industries and academics deal with a considerable amount of data and perform analysis on terabytes and even petabytes of data. To access their desired result, they use the Hadoop ecosystem and MapReduce to distribute work around various clusters (Dubey et al., 2015). This project is based on a stock market prediction based on Hadoop. They use Hive commands to create Hive tables to load data.

This is the era of technology, only few people use newspapers and other old media for trading on the stock exchange. Because of mobile technologies, users can directly buy or sell their shares from the online stock market. Also, users get every second update through their mobile (Jose et al., 2019). Hence, investors also used these technologies to discuss trade, market status, and dealing with security issues. This type of data is collected in the form of big data. Similarly, when planes fly, they keep transmitting data to headquarters or airbases. The air traffic control uses this data to track and monitor the current position and status of the flight. All this information is processed on a real-time basis. Since multiple air crafts transmit data regularly, the amount of transmitted data received by the flight controller is enormous, and it is accumulated in a vast volume within a concise time (Barros and Couto, 2013). It is a very challenging task to manage and process this massive amount of data called big data. In this study, the researcher demonstrates the methods to process this type of data.

Hundreds or even thousands of airline flights are canceled every year, which costs more money to passengers and owners. Many airlines are canceled due to bad weather conditions. Using Hadoop and MapReduce, the historical prediction can be maintained, predicting the delay and cancelation of a flight from historical data of weather and airlines (Patgiri et al., 2020). The historical dataset was taken to perform operations using pig and MapReduce, which produce output predictions based on temperature, snowfall, lousy weather, and many other factors. It also predicts the influence of cost due to delays and the cancelation of a flight. A model is proposed that determines the total number of flights canceled during 2012–2014, and their analysis is broken into months of each year. Researchers also analyzed the results of all flights diverted during each month of the year between 2012 and 2014.

The trend analysis is also analyzed for e-commerce websites. Using this project, we can easily find the trend of fashions, technologies, and music that varies from one geographical location to another (Satish and Kavya, 2017). Through trend analysis, companies can think of new products based on the needs of the customer, and they can do good strategic planning based on these trends. Amazon is one of the big e-commerce websites where people worldwide visit and see newly added products (Kaushik et al., 2018). The trend analysis is used to check the upcoming events all over the world. New trends come in fashion, living standards, traveling through cars, and many more. Hadoop is used to analyze this trend in this project and depending on these trends and upcoming events, new products were added. The search keywords from Google were taken and analyzed using Hadoop for finding occasional and even periodic events. Through these analyses, it is important to increase sales and attract an audience. This project will focus on data generated from Dailymotion for data mining and processing to make decisions to check their product market value. To accomplish this target, Hadoop, the distributed file system, is used.

The Hive is utilized to analyze temperature data and apply processing on 800,000 records (Lydia and Swarup, 2016). This analysis is done through the Hive query language, shortly, called HQL commands. This project supports in applying HQL commands for analyzing the data. Some of the common commands which are used are given below. Apache has implemented MapReduce, which is very time-consuming because of needed skills in programming languages, such as Jave (Lydia et al., 2016). The social media platform implemented Hive for its query-based features and similarity with SQL commands. For Warehousing projects, Hive is highly recommended (Capriolo et al., 2012; Salehi and Bernstein, 2018). With an increase in working remotely today, people worldwide can more easily work in one team, allowing multiple experts from different fields and domains, where they can input different types of data. MapReduce has no built-in support of the iterative type of programs; whereas Hadoop allows for processing iterative type of programs and applications from the Hadoop Program without any modification (Paul et al., 2016).

## Proposed Framework

**FIGURE 1:**
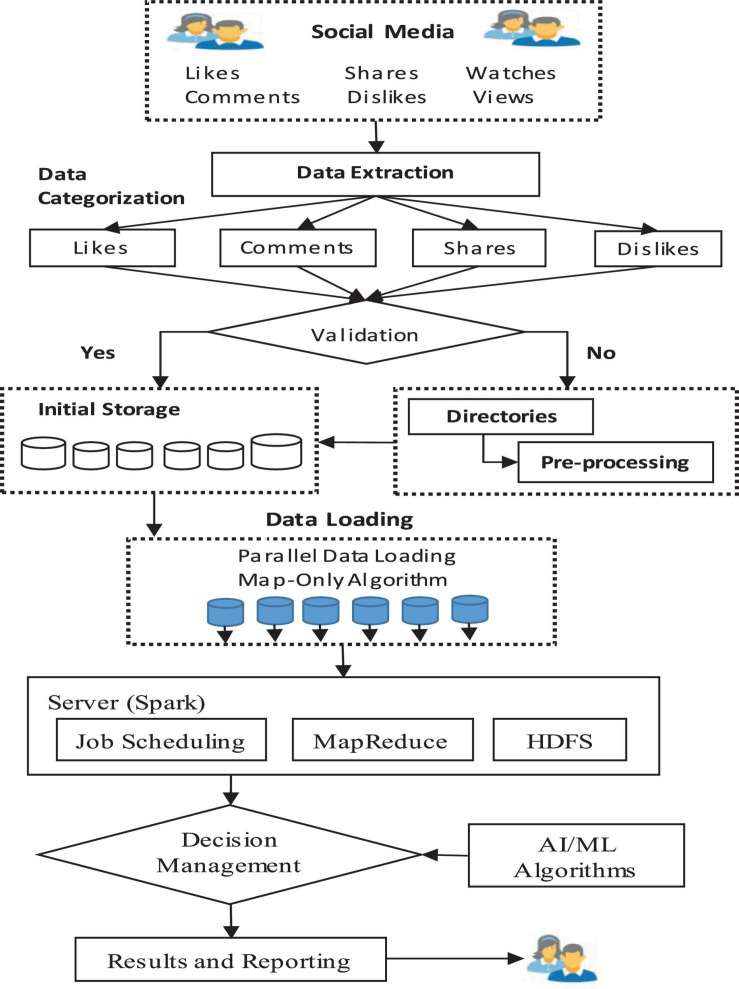
Overview of the proposed framework.

**FIGURE 2:**
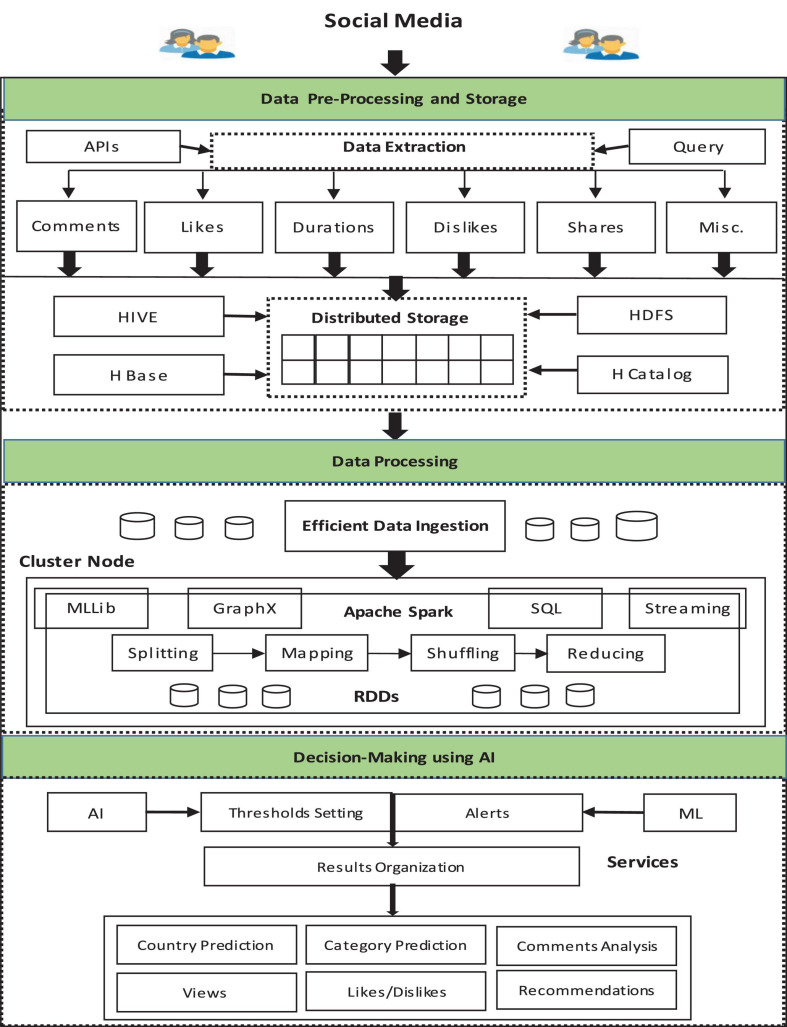
Proposed architecture.

An overview of the proposed framework is given in Figure 1. The framework is a parallel and distributed framework. Initially, the data related to a particular video is extracted and the video is scraped. The extracted data is recorded and aggregated in a specific format. Initially, the dataset is checked for anomalies and perm pre-processing. Afterward, the data is loaded into the proposed system using the parallel mechanism to speed up the data ingestion process. The processing of data is carried out by using the Apache Spark framework. The processed data is further utilized for decision-making using machine learning and AI approaches. Finally, the report is provided for decision-making. The detailed architecture of the proposed framework is depicted in Figure 2. The proposed architecture is composed of three layers: data pre-processing and storage, data processing, and decision management. A detailed description of the different layers is provided in the upcoming section.

### Pre-processing and Data Storage

The application programming interface (API) of Dailymotion is utilized to extract data from a particular channel through a specific set of queries. This project focuses on fetching data of a particular channel of Dailymotion using its API. We use the Dailymotion developer console to get a unique access key for fetching Dailymotion public channel data. The data is extracted in the form of a CSV file. The CSV file contains all the information about the channel and videos on that channel. The data available in the CSV file contain several anomalies including noise, corrupt data, denormalize data, duplicate values, and null values. Therefore, there is a need for preprocessing techniques to remove the anomalies. The proposed framework utilized data cleaning, data transformation, data normalization, and data integration. The accuracy of information relies on the recognition and removal of meaningless data.

The noise identification is done before noise removal. The data cleaning includes the detection and rectification of the imprecise data. The normalization is used to transform variables in data into specific series. The transformation is performed by converting the format of available data into a suitable format of processing. The big data must be stored in a specific systematic mechanism to process it efficiently. The proposed architecture utilizes the Hadoop Distributed File System (HDFS) distributed storage mechanism to store huge and gigantic datasets. HDFS grips a huge quantity of data and offers access at ease. The big datasets are stored across many nodes to be processed in parallel.

The Hive storage mechanism is also utilized and integrated with HDFS. The reason for the utilization of the Hive storage is the compatibility of CSV files with Hive that makes the loading process easy. The data is initially extracted in the text file that is in the form of unstructured data. To process analysis techniques, specific delimiters on CSV files are defined to load into Hive. It also works as an interface for data warehousing of Apache Hadoop-based data. It is a data warehousing infrastructure developed on top of Hadoop that allows querying data for data analysis. The CSV data is converted to Optimized Row Columnar (ORC) data and then loaded into the Hive table. A Dailymotion data table is created with a specific set of required fields. The H-catalog is used as a table storage management tool that processes the Hive tabular data into the Hadoop application for processing. The H-catalog is built on top of Hive that incorporates Hive data definition. Hive enables users to treat a file as an Structured Query Language (SQL) table with rows and columns. It provides read and write interfaces for Hadoop technologies.

### Data Processing Using Parallel Framework

The data processing of huge datasets is the key module of the proposed model. An integrated approach is used to process the big data. Special storage techniques are taken into consideration for efficient processing. HDFS and Hive storage techniques are integrated to achieve optimal distributed storage. The Apache Spark parallel and distributed framework are applied for fast and real-time stream processing of big data. The programming paradigm utilized by Apache Spark is the MapReduce paradigm. The MapReduce is the rationale for parallel functional processing. The data is loaded into the Spark framework using a parallel mechanism (e.g., map-only algorithm). Apache Spark maps the complex queries with MapReduce jobs for simplifying the complex process. The queries of Spark can be mapped into the phases of the MapReduce framework. Spark SQL handles the selection operations. Spark is a master-slave architecture, and the overall cluster is managed by the Spark master node. The proposed Spark architecture processes the data based on Resilient Distributed Dataset (RDD). An RDD is a distributed collection and immutable that can be wrought on in parallel. The RDD includes an object and is produced by ingesting an external dataset. The data collected from billions of customers is utilized as an actionable metric to perform better decision-making and get more customer satisfaction. The input is categorized into region, likes, duration, etc. The regional data is then analyzed to check the views from different regions and countries. The detail of the viewer and watch time are noted for future decisions. The likes of each video are analyzed to check the interest of the viewer. The daily view and comments analytics are created by running the queries on imported data.

### Decision Management

The decision management layer is a bridge between the proposed architecture and the outer world. It utilizes AI and ML algorithms. The thresholds are set using AI to analyze the specific dataset. The users are alerted using the AI mechanism. Based on the output, companies decide the enhancement of their investment decision-making using AI. The decisions can be utilized to market the projects. The proposed system utilizes the Dailymotion data to market the products based on region, country, and even based on a particular interest of users. Companies can find the peak and slow time of their viewership through a share, view count, subscriber, and audience retention. The companies can also find the trending product at a particular time. The changing behavior of people can be an important insight of companies.

## Results and Discussion

This section describes the implementation detail and results. This project focuses on fetching data of a particular channel of Dailymotion using its API. We use the Dailymotion developer console to get a unique access key for fetching Daily motion public channel data. The data is extracted in the form of a CSV file. The CSV file contains all the information about the channel and videos. After getting the API key, the.Net (C#) console application can be developed for fetching information based on search criteria. A text file will be generated by using this program, which will then be loaded from HDFS into the Hive database. In this project, we fetch YouTube data of a specific channel using API. We used Google Developers Console and generated a unique access key required to fetch YouTube public channel data. Once the API key is generated, a.Net (C#) based console application is designed to use the Dailymotion API for fetching video information based on search criteria. The text file output generated from the console application is then loaded from the HDFS file into the Hive database. The user can directly interact with HDFS using various commands. The queries will be run on big data through Hive to get the required data. This data will then be used by management for analysis. Besides, Apache Spark 3.0 is utilized for real-time stream processing of big data. The pyspark library is used for the implementation of spark workers. The MLLib library is utilized for applying the Machine Learning (ML) algorithm in the spark context. The graphX library is utilized for graph implementation.

**FIGURE 3:**
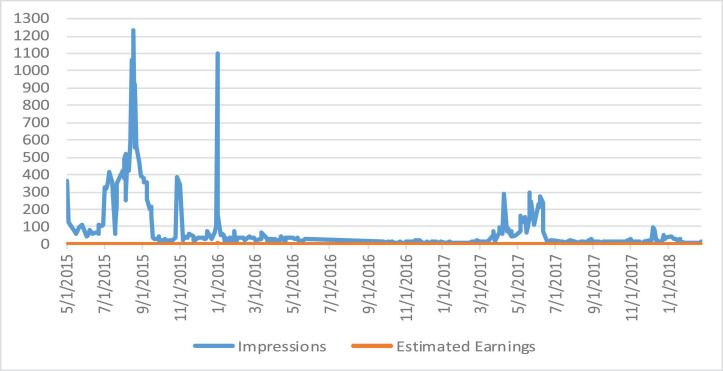
Impressions vs. estimated earnings.

**FIGURE 4:**
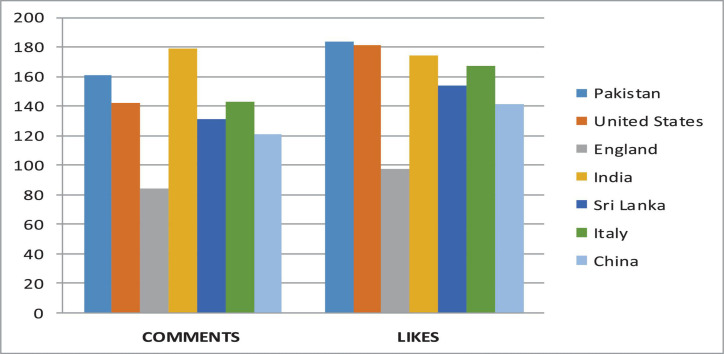
Country-wise comments and likes.

**FIGURE 5:**
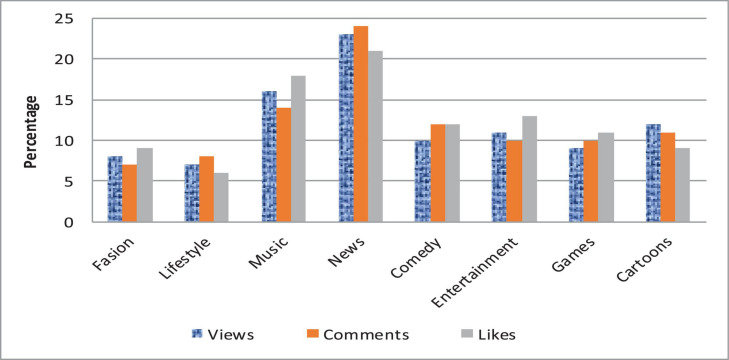
Category-wise detail.

We analyze the data and perform various operations to find the number of comments on the particular video and also the person who has uploaded the video. The dataset utilized contains the channel ID, category, duration, view count, comment count, like count, and country code. Dailymotion also provides video monetization options for its users, and most Dailymotion users have their channels with a monetized video that generates revenue for them through video ads. We extracted a CSV file from Dailymotion, and then uploaded it on Hadoop HDFS storage to analyze. The extracted file contains some meaningless information. The final file contains three columns: date, number of impressions, and earnings. We have generated the report of earnings within the particular time frame, and the detailed sum of an impression on a video is shown in Figure 3. The country-wise comments and like counts are shown in Figure 4. The category-wise detail of views, comments, and likes are illustrated in Figure 5.

**FIGURE 6:**
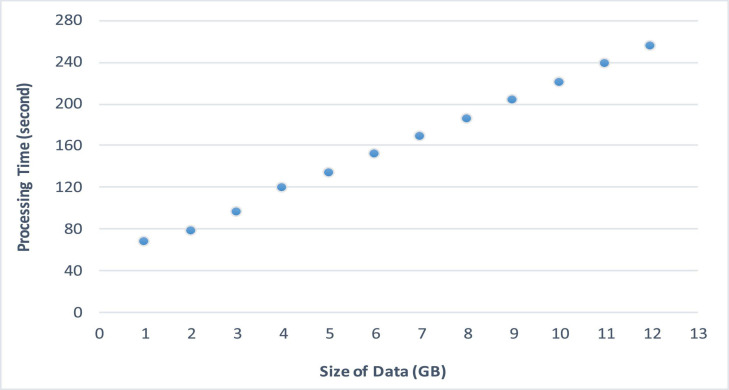
Processing time.

**FIGURE 7:**
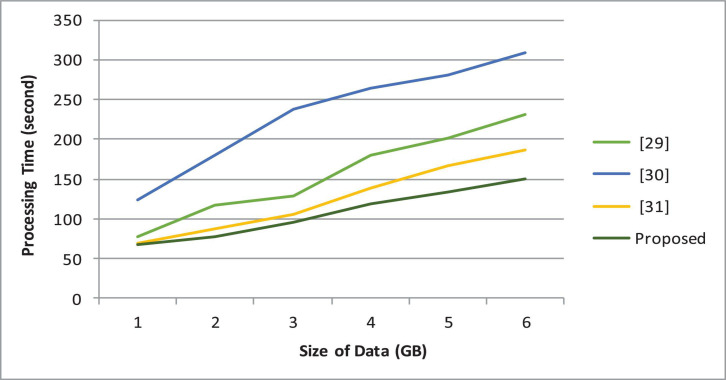
Comparative analysis.

Figure 6 demonstrates the processing time of the proposed architecture. Besides, the comparative analysis of the proposed architecture with state-of-the-art is demonstrated in Figure 7.

## Conclusion

The use of big data in the field of social media is essential. The organizations that use big data have a huge advantage over the one which is still practicing relational database techniques. These organizations better know the importance of big data than the one which has no big data implementation. This product is intended to show the data analysis of Dailymotion and some key results. This article proposed a model using Apache Spark. The proposed architecture is three-layered architecture. The main objective of this project is to demonstrate the use of Apache Spark parallel and distributed framework technologies with other storage and processing mechanisms. The effectiveness of the proposed architecture is also highlighted. In this way, many other features can be determined, and the company could know the details of its competitor and clients. If a company uploads its marketing video on Dailymotion, its video becomes more prominent than the base of views and likes.

## Data Availability Statement

The original contributions presented in the study are included in the article/supplementary material, further inquiries can be directed to the corresponding author.

## Author Contributions

MT: idea and logic. MB: writer, logic, and implementation. MP: supervision and review. AK: review and drafting. MA: review and implementation. SK: drafting and review. All authors contributed to the article and approved the submitted version.

## Conflict of Interest

The authors declare that the research was conducted in the absence of any commercial or financial relationships that could be construed as a potential conflict of interest.
